# Supplementary material for: Men’s Psychotherapy Use, Male Role Norms, and Male-Typical Depression Symptoms: Examining 716 Men and Women Experiencing Psychological Distress
Source: Behav Sci (Basel). 2021 Jun 2;11(6):83. doi: 10.3390/bs11060083 (PMC8228644; doi:10.3390/bs11060083)
Supplement: Supplementary file 1 [file behavsci-11-00083-s001.zip › behavsci-1202030-supplementary.pdf]

Men's Psychotherapy Use, Male Role Norms, and Male-Typical Depression Symptoms: Examining 716 Men and Women Experiencing Psychological Distress

Supplementary material

Content

Figure S1: *Distribution of Self-Assessed Dimensional Gender* *page 2*
Figure S2: *Interaction Effects in Female Participants* *page 3*
Table S1: *Group Comparisons for Heterosexual Male and Female Participants* *page 4*
Table S2: *Second-Order Partial Correlation Matrices for the applied Questionnaires* *page 5*
Table S3: *Summary of Binary Logistic Regression Models controlled for Covariates for Male Participants* *page 6*
Table S4: *Summary of Binary Logistic Regression Models controlled for Covariates for Female Participants* *page 7*

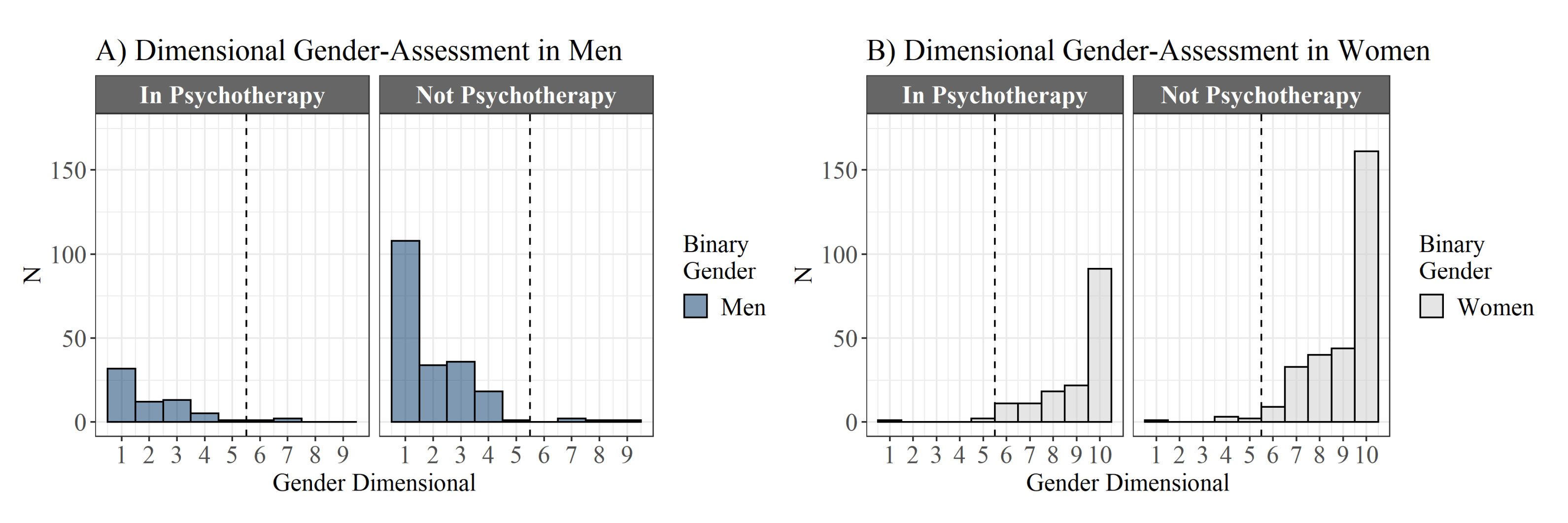

Figure S1. Distribution of Self-Assessed Dimensional Gender

Note. *N* = Number of participants. 1 = masuline pole, 10 = feminine pole. The vertical dashed line indicates the middle of the scale (above = more towards femininity pole; below = more towards masculinity pole).

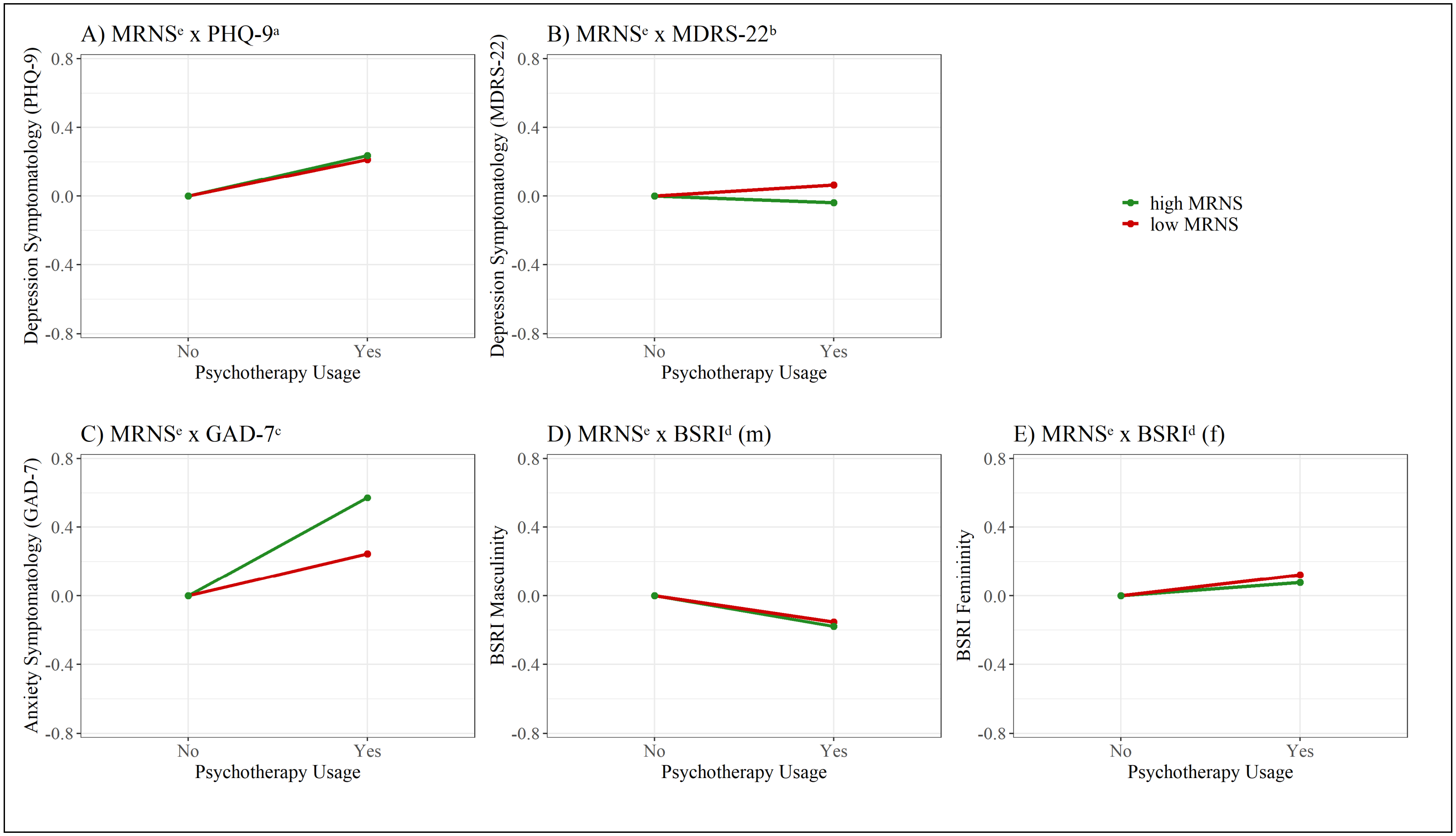

Figure S2. Interaction Effects in Female Participants

Note. x = interaction, *m* = masculine subscale, *f* = feminine subscale. The questionnaire values on the y-axis were *z*-standardized and linearly transformed to have a joint reference point a y = 0

[a] PHQ-9 = Patient Health Questionnaire - 9

[b] MDRS-22 = Male Depression Risk Scale - 22

[c] GAD-7 = Generalized Anxiety Disorder - 7

[d] BSRI = Bem Sex Role Inventory

[e] MRNS = Male Role Norm Scale

Table S1. Group Comparisons for Heterosexual Male and Female Participants

	PHQ-9[a]			MDRS-22[b]			GAD-7[c]		
	M *(SD)*	t *(df)*	*p*	M *(SD)*	t *(df)*	*p*	M *(SD)*	t *(df)*	*p*
**Men in PT**	12.4 *(6.2)*	−1.48 *(186)*	.630	30.3 *(20.0)*	−2.73 *(186)*	0.242	10.9 *(5.1)*	1.68 *(186)*	0.094
**Men not in PT**	12.9 *(5.6)*			34.3 *(19.8)*			9.5 *(4.8)*		
**Women in PT**	15.4 *(5.6)*	2.02 *(277)*	**0.045***	32.2 *(18.7)*	0.15 *(277)*	0.0878	12.9 *(3.9)*	4.3 *(277)*	**<0.001*****
**Women not in PT**	14.0 *(5.3)*			31.8 *(16.6)*			10.5 *(3.9)*		

	BSRI[d] (m)			BSRI[d] (f)			MRNS[e]		
	M *(SD)*	t *(df)*	*p*	M *(SD)*	t *(df)*	*p*	M *(SD)*	t *(df)*	*p*
**Men in PT**	55.0 *(11.5)*	−2.12 *(100.1)*	**0.037***	68.8 *(13.7)*	0.49 *(186)*	0.626	73.5 *(31.4)*	−0.98 *(186)*	0.326
**Men not in PT**	59.6 *(16.2)*			67.6 *(14.0)*			78.3 *(27.1)*		
**Women in PT**	54.2 *(14.4)*	−1.70 *(277)*	0.094	74.9 *(11.8)*	1.13 *(277)*	0.259	58.1 *(17.3)*	−1.30 *(277)*	0.194
**Women not in PT**	57.4 *(14.7)*			73.2 *(11.6)*			61.7 *(22.8)*		

Note. *M* = mean score, *SD* = standard deviation, *t* = t-value, *df* = degrees of freedom, *p* = *p*-value
[a] PHQ-9 = Patient Health Questionnaire - 9
[b] MDRS-22 = Male Depression Risk Scale - 22
[c] GAD-7 = Generalized Anxiety Disorder - 7
[d] BSRI = Bem Sex-Role Inventory
[e] MRNS = Male Role Norm Scale
* = $p < 0.05$, ** = $p < 0.01$, *** = $p < 0.001$

Table S2. Second-Order Partial Correlation Matrices for the applied Questionnaires

A) Male Participants							
Variable	*M*	*SE*	**PHQ-9[a]**	**MDRS-22[b]**	**GAD-7[c]**	**BSRI[d] (m)**	**BSRI[d] (f)**
**PHQ-9[a]**	13.7	6.0					
**MDRS-22[b]**	33.7	19.7	**0.53*****				
**GAD-7[c]**	10.3	5.0	**0.65*****	**0.51*****			
**BSRI[d] (m)**	57.8	14.7	**−0.31*****	−0.12	**−0.19****		
**BSRI[d] (f)**	67.9	13.3	−0.08	**−0.16***	−0.01	**0.30*****	
**MRNS[e]**	73.2	26.7	0.09	**0.29*****	0.12	**0.12***	**−0.22*****
**B) Female participants**							
**PHQ-9[a]**	14.8	5.4					
**MDRS-22[b]**	32.0	17.1	**0.56*****				
**GAD-7[c]**	11.6	4.5	**0.63*****	**0.46*****			
**BSRI[d] (m)**	55.7	14.0	**−0.30*****	**−0.12***	**−0.21*****		
**BSRI[d] (f)**	73.4	11.3	−0.08	−0.02	0.05	**0.25*****	
**MRNS[e]**	57.7	20.1	−0.03	0.05	−0.05	0.04	**−0.14****

Note. m = masculine subscale, *f* = feminine subscale, *M* = mean value, *SE* = standard error. Partial correlations were controlled for age and educational level.
[a] PHQ-9 = Patient Health Questionnaire—9
[b] MDRS-22 = Male Depression Risk Scale—22
[c] GAD-7 = Generalized Anxiety Disorder—7
[d] BSRI = Bem Sex-Role Inventory
[e] MRNS = Male Role Norm Scale
* = $p < 0.05$, ** = $p < 0.01$, *** = $p < 0.001$

Table S3. Summary of the Binary Logistic Regression Models controlled for Covariates for Male Participants

	**No interaction**		**MRNS x PHQ-9**		**MRNS x MDRS-22**		**MRNS x GAD-7**		**MRNS x BSRI (m)**		**MRNS x BSRI (f)**	
Predictor	β *(SE)*	*p*	β *(SE)*	*p*	β *(SE)*	*p*	β *(SE)*	*p*	β *(SE)*	*p*	β *(SE)*	*p*
**Intercept**	0.12 *(1.40)*	0.930	4.17 *(1.89)*	**0.028***	2.02 *(1.60)*	0.207	2.18 *(1.65)*	0.186	−2.35 *(2.02)*	0.244	−3.24 *(2.33)*	0.164
**PHQ-9**[a]	−0.23 *(0.34)*	0.505	−2.72 *(0.84)*	**0.001****	−0.18 *(0.35)*	0.609	−0.15 *(0.35)*	0.660	−0.24 *(0.34)*	0.481	−0.20 *(0.34)*	0.558
**MDRS-22**[b]	−0.71 *(0.25)*	**0.004****	−0.77 *(0.26)*	**0.002****	−2.16 *(0.63)*	**<0.001*****	−0.68 *(0.25)*	**0.006****	−0.71 *(0.25)*	**0.004****	−0.73 *(0.25)*	**0.004****
**GAD-7**[c]	0.85 *(0.31)*	**0.006****	0.99 *(0.32)*	**0.002****	1.00 *(0.32)*	**0.002****	−0.72 *(0.70)*	0.301	0.81 *(0.31)*	**0.009****	0.84 *(0.31)*	**0.007****
**BSRI**[d] **(m)**	−0.44 *(0.19)*	**0.021***	−0.37 *(0.20)*	0.067	−0.42 *(0.19)*	**0.029***	−0.39 *(0.19)*	**0.044***	0.29 *(0.47)*	0.533	−0.46 *(0.19)*	**0.015***
**BSRI**[d] **(f)**	0.04 *(0.19)*	0.823	*0.03 (0.20)*	0.893	0.06 *(0.20)*	0.774	0.07 *(0.20)*	0.732	0.03 *(0.20)*	0.868	0.82 *(0.48)*	0.083
**MRNS**[e]	−0.02 *(0.17)*	0.902	−1.53 *(0.52)*	**0.003****	−0.82 *(0.36)*	**0.021***	−1.02 *(0.45)*	0.024*	0.97 *(0.61)*	0.113	1.19 *(0.69)*	0.086
**Age**	0.01 *(0.01)*	0.427	0.01 *(0.02)*	0.705	0.01 *(0.02)*	0.425	0.01 *(0.01)*	0.459	0.01 *80.01)*	0.592	0.01 *(0.01)*	0.411
**Relationship**	0.03 *(0.14)*	0.854	0.06 *(0.15)*	0.704	0.03 *(0.15)*	0.863	0.04 *(0.15)*	0.809	0.06 *(0.15)*	0.688	0.04 *(0.14)*	0.777
**Sex. Orient.**	0.09 *(0.15)*	0.552	0.11 *(0.15)*	0.449	0.09 *(0.15)*	0.566	0.12 *(0.15)*	0.427	0.08 *(0.15)*	0.569	0.10 *(0.15)*	0.482
**Education**	−0.03 *(0.09)*	0.757	−0.07 *(0.15)*	0.427	−0.04 *(0.09)*	0.690	−0.03 *(0.09)*	0.783	−0.03 *(0.09)*	0.740	−0.02 *(0.09)*	0.864
**Interaction**			0.93 *(0.29)*	**0.001****	0.47 *(0.18)*	**0.011***	0.58 *(0.24)*	**0.015***	−0.27 *(0.17)*	0.097	−0.30 *(0.16)*	0.073
Omnibus statistics												
**χ^2 (*df*)**	21.70 *(10)*		33.22 *(11)*		29.14 *(11)*		27.88 *(11)*		24.67 *(11)*		24.89 *(11)*	
***p* (omnibus)**	**0.017***		**<0.001*****		**0.002****		**<0.003****		**0.010***		**0.009****	
**Pseudo R^2 (Nagelkerke)**	0.116		0.174		0.154		0.147		0.131		0.132	

Note. x = interaction, *m* = masculine subscale, *f* = feminine subscale, β = estimated regression coefficient, *SE* = standard error, *p* = *p*-value. All calculations were controlled for age (*Age*), relationship status (*Relationship*), sexual orientation (*Sex. Orient.*) and education level (*Education*).
[a] PHQ-9 = Patient Health Questionnaire—9
[b] MDRS-22 = Male Depression Risk Scale—22
[c] GAD-7 = Generalized Anxiety Disorder—7
[d] BSRI = Bem Sex-Role Inventory
[e] MRNS = Male Role Norm Scale
* = $p < 0.05$, ** = $p < 0.01$, *** = $p < 0.001$

Table S4. Summary of the Binary Logistic Regression Models controlled for Covariates for Female Participants

	**No interaction**		**MRNS x PHQ-9**		**MRNS x MDRS-22**		**MRNS x GAD-7**		**MRNS x BSRI (m)**		**MRNS x BSRI (f)**	
Predictor	β *(SE)*	*p*	β *(SE)*	*p*	β *(SE)*	*p*	β *(SE)*	*p*	β *(SE)*	*p*	β *(SE)*	*p*
**Intercept**	−1.99 *(1.12)*	0.076	−3.40 *(1.37)*	**0.013***	−3.66 *(1.29)*	**0.005****	−2.23 *(1.41)*	0.116	−1.37 *(1.86)*	0.461	−1.74 *(2.63)*	0.509
**PHQ-9[a]**	0.09 *(0.25)*	0.728	1.06 *(0.61)*	0.082	0.06 *(0.25)*	0.814	0.08 *(0.25)*	0.743	0.09 *(0.25)*	0.716	0.09 *(0.25)*	0.730
**MDRS-22[b]**	−0.32 *(0.17)*	0.061	−0.31 *(0.17)*	0.065	1.02 *(0.56)*	**0.068***	−0.32 *(0.17)*	0.060	−0.32 *(0.17)*	0.060	−0.32 *(0.17)*	0.060
**GAD-7[c]**	0.80 *(0.22)*	**<0.001*****	0.82 *(0.22)*	**<0.001*****	0.82 *(0.22)*	**<0.001*****	0.96 *(0.64)*	.131	0.80 *(0.22)*	**<0.001*****	0.80 *(0.22)*	**<0.001*****
**BSRI[d] (m)**	−0.11 *(0.12)*	0.350	−0.13 *(0.13)*	0.302	−0.13 *(0.13)*	0.295	−0.12 *(0.12)*	0.346	−0.28 *(0.41)*	0.496	−0.12 *(0.12)*	0.354
**BSRI[d] (f)**	0.19 *(0.15)*	0.199	0.18 *(0.15)*	0.230	0.20 *(0.15)*	0.190	0.19 *(0.15)*	0.215	0.19 *(0.15)*	0.200	0.14 *(0.52)*	0.791
**MRNS[e]**	−0.20 *(0.15)*	0.173	0.38 *(0.34)*	0.274	0.46 *(0.28)*	0.109	−0.10 *(0.38)*	0.793	−0.43 *(0.57)*	0.454	−0.29 *(0.86)*	0.739
**Age**	−0.01 *(0.01)*	0.675	−0.01 *(0.01)*	0.735	−0.01 *(0.01)*	0.749	−0.01 *(0.01)*	0.678	−0.01 *(0.01)*	0.679	−0.01 *(0.01)*	0.673
**Relationship**	0.07 *(0.11)*	0.501	0.07 *(0.11)*	0.519	0.08 *(0.11)*	0.481	0.07 *(0.11)*	0.504	0.07 *(0.11)*	0.500	0.07 *(0.11)*	0.501
**Sex. Orient.**	0.02 *(0.09)*	0.852	0.01 *(0.09)*	0.868	0.01 *(0.09)*	0.929	0.02 *(0.09)*	0.859	0.01 *(0.09)*	0.868	0.02 *(0.09)*	0.850
**Education**	0.05 *(0.07)*	0.520	0.04 *(0.08)*	0.612	0.03 *(0.07)*	0.653	0.05 *(0.07)*	0.525	0.05 *(0.07)*	0.527	0.05 *(0.07)*	0.521
**Interaction**			−0.37 *(0.21)*	0.078	−0.49 *(0.20)*	0.130	−0.06 *(0.21)*	0.784	0.06 *(0.14)*	0.675	0.02 *(0.18)*	0.916
Omnibus statistics												
**χ^2 (*df*)**	30.88 *(10)*		33.83 *(11)*		37.41 *(11)*		30.96 *(11)*		31.06 *(11)*		30.90 *(11)*	
***p* (omnibus)**	**<0.001*****		**<0.001*****		**<0.001*****		**0.001****		**0.001****		**0.001****	
**pseudo R^2 (Nagelkerke)**	0.092		0.100		0.110		0.092		0.092		0.092	

Note. x = interaction, *m* = masculine subscale, *f* = feminine subscale, β = estimated regression coefficient, *SE* = standard error, *p* = *p*-value. All calculations were controlled for age (*Age*), relationship status (*Relationship*), sexual orientation (*Sex. Orient.*) and education level (*Education*).

[a] PHQ-9 = Patient Health Questionnaire - 9

[b] MDRS-22 = Male Depression Risk Scale - 22

[c] GAD-7 = Generalized Anxiety Disorder - 7

[d] BSRI = Bem Sex-Role Inventory

[e] MRNS = Male Role Norm Scale

* = $p < 0.05$, ** = $p < 0.01$, *** = $p < 0.001$
